# The fear in desire: linking desire thinking and fear of missing out in the social media context

**DOI:** 10.1186/s40359-023-01216-0

**Published:** 2023-06-03

**Authors:** Annika Brandtner, Elisa Wegmann

**Affiliations:** Department of General Psychology: Cognition and Center for Behavioral Addiction Research (CeBAR), University of Duisburg-Essen, Forsthausweg 2, LE220, 47057 Duisburg, Germany

**Keywords:** Desire thinking, FoMO, Craving, Problematic SNS use, Elaborated intrusion theory

## Abstract

According to the Elaborated Intrusion Theory of Desire, desire thinking and an associated deficit are fundamental factors to the emergence of craving. In the special case of problematic social networking sites (SNS) use, this experienced deficit could be constituted of an online-specific fear of missing out (FoMO). To test the interaction of these cognitions and their influence on problematic SNS use, we tested a serial mediation model on a sample of *N* = 193 individuals who use SNS (73% female, *M*_*age*_ = 28.3, *SD* = 9.29). We found that desire thinking predicted FoMO and both variables were only significant predictors of problematic SNS use when considered in interplay with craving. Ad hoc analyses revealed that the verbal subcomponent of desire thinking is more strongly associated with FoMO than imaginal prefiguration. Our results highlight that neither desire thinking nor FoMO are inherently dysfunctional but become problematic when they increase craving for potentially problematic SNS use.

## Background

The use of social networking sites (SNS) has become an integral part of our lives. We can access them anywhere and anytime. For some, this feels like time well-spent on helpful or enriching activities on SNS. However, a vulnerable minority feels guilty about this wasted time [1], experiences a decrease in well-being [2, 3], or reports that they lose control over their use [4, 5]. While there is a debate on whether we are over-pathologizing everyday behaviors [6], a line of research claims that if the use of SNS takes precedence over other life activities, becomes hard to control, and is continued despite the occurrence of negative consequences, it might resemble addictive behavior patterns. Main lines of reasoning that certain behaviors might be considered as addictive refer to (1) the scientific evidence for the clinical relevance of the behavior, (2) the theoretical embedding of phenomena, and (3) the similarity of underlying cognitive and affective mechanisms to those of substance use disorders [7]. Addiction-specific mechanisms that have therefore come into focus of research on the use of SNS are cue-reactivity and craving (e.g., [8-11]).

There is a broad variety of craving conceptualizations and theories, including conditioning models, psychobiological models, motivational models, and cognitive models (for review, see [12]). All of them aim at explaining the same phenomenon: a strong and irresistible desire for an appetitive object, state, or activity (e.g., [13, 14]). A theory that shows transdiagnostic validity in the phenomenology of craving for a variety of appetitive substances such as alcohol, food, soft drinks, and tobacco, is the Elaborated Intrusion Theory of Desire (EIT; [15-17]). More recently, also gambling cravings have been explored through the eyes of the EIT, having led to the application of this theory also in the context of addictive behaviors [18]. In its basic assumptions, the EIT postulates an interplay of cue-elicited automatic associations and the effortful cognitive elaboration of those associations that have intruded into awareness [15]. The process that is responsible for the elaboration of an initial intrusion of an appetitive target is desire thinking [19], which is constituted by the human ability to process future-oriented thinking [20, 21]. Thus, desire thinking refers to an imaginal prefiguration and verbal preoccupation that is voluntary and effortful [19, 22, 23]. Desire thinking has been found to be more pronounced in individuals with addictive behaviors (e.g., [24, 25]) and has more recently been theoretically embedded in an extended version of the I-PACE model for specific Internet-use disorders [26, 27]. More specifically, evidence accumulates that desire thinking might also be associated with problematic SNS use [28-31]. In summary, desire thinking is not inherently dysfunctional since it resembles, according to respective theoretical considerations [19], a human ability to foresee and elaborate future events. However, in the context of desires and cravings for possibly unwanted behaviors, desire thinking might be ascribed a more problematic role and function [32, 33].

Desire thinking is assumed to have a strong motivational power through two self-reinforcing loops in the EIT [15]. First, it is assumed that desire thinking elicits a feeling of pleasure and relief in itself, also referred to as urge [34], since the imagery component is strongly associated with the experience of emotions [35]—which is a transdiagnostic feature of imagery in desire and psychopathologies in general [36]. Second, since the current and the desired state are weighed against each other, the conclusion might be drawn that there is a gap between reality and imagination. This gap might manifest as the experience that “something is missing”, which is called a sense of associated deficit in the EIT [15]. In the special case of SNS use, this sense of deficit, or the feeling that something is missing, could be determined by the experience that one does not know what happens on social media at the very moment. That is, the sense of deficit might be constituted of a lack of knowledge and the urge of wanting to know what is going on online. In the context of SNS use, this state of experienced information deficit might be closely linked to the concept of fear of missing out (FoMO; [37]).

FoMO is conceptualized as “a pervasive apprehension that others might be having rewarding experiences from which one is absent” and “a desire to stay continually connected with what others are doing” ([37], p. 1841). Traditionally, FoMO was conceptualized within an application of the self-determination theory where social relatedness is one basic human need that needs to be fulfilled to experience psychological well-being and employ intrinsic motivation [37, 38]. Besides its understanding as being a general human need, FoMO has repetitively been brought into relation with problematic SNS use in form of a technology-related cognition (for review and meta-analysis, see [39, 40]). That is, being on SNS might be used to satisfy the need for social relatedness, especially when individuals experience increased social isolation as is sometimes the case in depressive and anxious individuals [41, 42]. However, the experience of a need or even a fear might not be enough to explain a problematic SNS. Mechanisms by means of which FoMO could exert its effect on problematic SNS use might include unrealistic use expectancies of the SNS use [42], sensitivity to social-media-related stress [43], and the SNS use intensity and frequency [42, 44]. In addition, Wegmann et al. [45] suggest distinguishing a general *trait FoMO* variable from a variable that focuses more on the SNS-related context. The latter describes the extent to which online-related expectations, fears, and reward mechanisms are specific to SNS use and is defined as *online FoMO*. Integrating the above made theoretical considerations, online FoMO could be a product of desire thinking and thus be a constituting factor in the emergence of craving. That is, examining the relationships between desire thinking, FoMO, and craving in predicting problematic SNS use could help to understand in what way desire thinking might constitute a risk factor for problematic SNS (e.g., [28, 30, 46]). Creating awareness for such associations not only feeds theories for a better understanding of cognitive and affective mechanisms, but can also transfer into clinical practice where practitioners get sensitized for such fine-grained stringing together of mental processes.

**Fig. 1:**
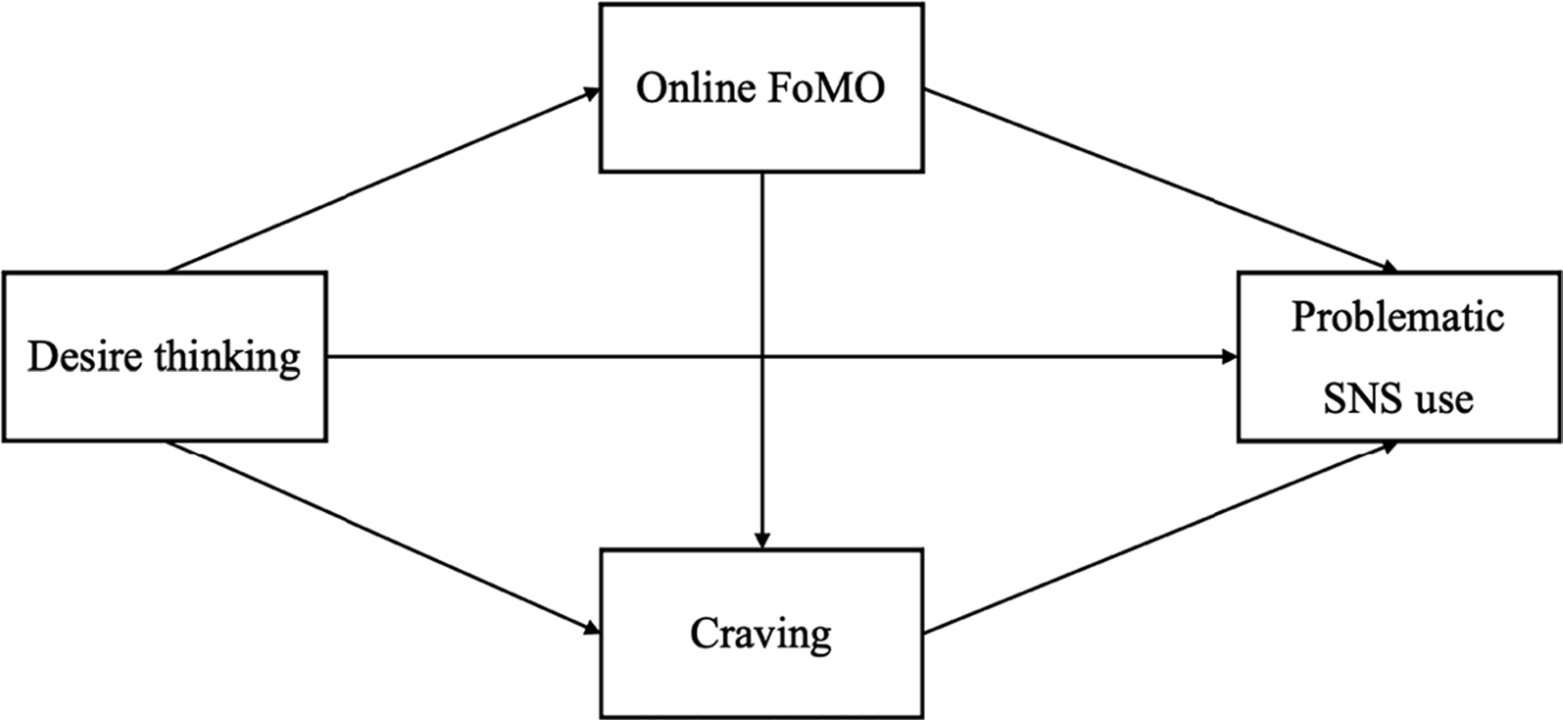
Hypothesized model of the mediating effect of FoMO and craving in the relation between desire thinking and problematic SNS use. *Note*. Operationalizations of variables are described in the methods section

In summary, according to the EIT [15] and the extended I-PACE model around desire thinking [27], engaging in desire thinking could provoke an experienced sense of associated deficit which, in the context of SNS use, might be constituted by an online-specific FoMO. This subjectively experienced deficit might be responsible for a heightened craving to use SNS. Thus, not desire thinking or FoMO alone, but their interplay with craving might eventually explain an uncontrolled and problematic use of SNS (see Fig. 1).

## Methods

### Participants

A total of *N* = 246 participants were recruited to complete an online survey. The cleaned sample (see statistical analyses) consisted of *n* = 193 participants (141 female, 4 non-binary), aged between 18 and 70 years (*M* = 28.3, *SD* = 9.29). The community sample was collected between May and July 2022 where 51% indicated to be students at a German University, 39% to be fully or part-time employed, and the rest being in training, retired, or other.

### Instruments

#### Desire thinking

The Desire Thinking Questionnaire (DTQ; [23]) was used in its German translation [47] to assess the tendency to engage in desire thinking around the use of SNS. Five items were answered for the subcomponent imaginal prefiguration (e.g., “I imagine myself using social networks”) and five items were answered for the subcomponent verbal perseveration (e.g., “I repeat mentally to myself that I need to use social networks”) on a four-point Likert scale ranging from 1 = *almost never* to 4 = *almost always*. Higher sum scores indicate more frequent desire thinking. The Cronbach’s alpha = 0.843 of the overall sum score indicates a good internal consistency in this sample.

#### Fear of missing out

The Fear of Missing Out Scale [37] was used in an adapted version [45] that can assess an online-specific FoMO with seven items (e.g., “I'm afraid of not being up to date on my social networks”) next to a general trait-like FoMO with five items (e.g., “I feel insecure when I do not know what my friends are up to”). The items were answered on a five-point Likert scale ranging from 1 = *completely disagree* to 5 = *completely agree* with higher mean scores indicating a higher expression of FoMO. Only the online-specific FoMO subscale was used for hypotheses and analyses in this study. A Cronbach’s alpha of 0.813 indicates a good internal consistency of the subscale in this sample.

#### Craving

The Craving Experience Questionnaire (CEQ; [48]) was used in its adaptation for addictive behaviors (i.e., gambling; [49]) and adapted to the use of SNS. Three subscales with three items each measure the intrusiveness of thoughts (e.g., “How often is it difficult for you not to think about social networks?”), the intrusiveness of imagery (e.g., “How often do you imagine the sound of using social networks?”), and the intensity of the accompanying urge (e.g., “How often do you experience a strong urge to use social networks?”) on a 11-point Likert scale from 0 = *not at all* to 10 = *extremely often*. Higher mean scores indicate a higher frequency of craving experiences. In this sample, the overall mean score was used, and the Cronbach’s alpha of 0.898 indicates an excellent internal consistency.

#### Symptom severity of problematic SNS use

The Assessment of Criteria for Specific Internet-use Disorders (ACSID-11; [50]) was used to assess problematic SNS use. The scale assesses, on three subscales with three items each, the ICD-11 criteria for addictive behaviors, that is, impaired control over the use, the priority given to the use, and the escalation or continuation despite negative consequences (e.g., “In the past 12 months, have you continued or increased the activity even though it has caused you physical or mental complaints/diseases?”). Additionally, two items assess the marked distress and the functional impairment due to the use. All items were rated on a four-point Likert scale ranging from 0 = *never* to 3 = *often*. A higher mean score indicates a higher frequency of experiencing the symptoms. In addition, the items were also answered on another scale from 0 = *not at all intense* to 3 = *intense* to capture the intensity of the ICD-11 criteria. However, this scale was not considered in the present analysis. Internal consistency of the frequency mean score was excellent with Cronbach’s alpha = 0.864.

### Procedure

The procedure was approved by the local ethics committee and the study was performed in line with the principles of the Declaration of Helsinki. The study was a mere online study with two parts. The first part contained an online experimental manipulation of desire thinking and its effect on state variables (i.e., participants in the experimental condition were prompted—by written instructions—to imagine a typical situation where they use SNS whereas a control group imagined a walk through a forest; both groups were tasked to type in their imagined scenario into open text fields; results not being published due to error-prone online manipulation). The following analyses are controlled for a possible influence of this manipulation, indicating no significant effects (see Table 1). The second part involved the questionnaires to be analyzed in this study. Participants started by giving informed consent on the study protocol which was presented to them on the first page. After the experimental paradigm, they proceeded to the questionnaires that were presented in the order of the theoretically assumed seriality. After disclosing demographic information, participants answered quality check queries (i.e., if they used social media while answering the survey, if we should use their data) and were finally provided with a debriefing, explaining the purpose of the study and an option to leave a comment.

**Table 1:** Descriptive statistics of study variables

	Descriptive statistics			Correlations				
	*M* (*SD*)	Range	Independent t-test^a^	DTQ-IP	DTQ-VP	FoMO	CEQ	ACSID-11
DTQ	16.05 (4.77)	10–30	*p* = .309	.901***	.917***	.568***	.824***	.627***
DTQ-IP	7.98 (2.51)	5–15	*p* = .092	–	.654***	.451***	.716***	.551***
DTQ-VP	8.06 (2.73)	5–17	*p* = .820		–	.578***	.781***	.589***
FoMO	2.24 (0.79)	1.00–4.29	*p* = .206			–	.692***	.518***
CEQ	2.15 (1.60)	0.00–6.89	*p* = .243				–	.705***
ACSID-11	0.75 (0.54)	0.00–2.27	*p* = .723					–

*N* = 193; *DTQ* desire thinking questionnaire, *IP* imaginal prefiguration subscale of DTQ, *VP* verbal perseveration subscale of DTQ, *FoMO* fear of missing out scale, *CEQ* craving experience questionnaire, *ACSID-11* assessment of criteria for specific internet-use disorders

^a^Independent *t*-tests were calculated for the questionnaires between conditions prior to an experimental manipulation (first part of the study, not reported) indicating that study variables were not influenced by the manipulation; ***p < .001

### Statistical analyses

The data sample was cleaned before statistical analyses. Participants were excluded if they fulfilled one or more of six criteria, which were the indication in the binary quality-check query that we should not use their data, that the mother language not was German, the indication that they used SNS during the survey, LongString analyses (i.e., having scored the same in all items of more than half of the questionnaires), time needed for the survey (i.e., if it took them three times longer than the average to complete the survey), or a significant Mahalanobis distance of relevant study variables. This led to the exclusion of 53 data sets, resulting in *n* = 193 participants. The questionnaires to be used in this study were not affected by experimental manipulation as indicated by non-significant *t*-tests between experimental conditions (see Table 1). Descriptive statistics and correlational analyses were conducted with SPSS v27, path analyses were conducted using MPlus8 v1.7, on a MacBook Pro running Monterey v12.4. Goodness-of-fit of the path model was considered satisfactory with a standard root mean square residual (SRMR) below 0.08, root mean square error of approximation (RMSEA) below 0.08, comparative fit indices (CFI/TLI) above 0.90, and if the degrees of freedom ratio (χ^2^/df) was below 3 [51, 52].

## Results

### Descriptive statistics

In a multiple-choice query, 55% of the participants indicated to use Facebook, 77% Instagram, 98% WhatsApp, 31% TikTok, 34% Snapchat, 17% Twitter, 14% Reddit, 31% Pinterest, 78% YouTube, 34% LinkedIn, 19% Telegram, and 8% indicated other applications in an open textbox (e.g., Discord, Signal, Twitch, Threema, Jodel, Xing). For applications they indicated to use, participants reported to use Facebook averagely 23.81 min (*SD* = 29.69), Instagram 64.38 min (*SD* = 54.95), WhatsApp 71.62 min (*SD* = 104.78), TikTok 68.78 min (*SD* = 86.1), Snapchat 34.53 min (*SD* = 102.55), Twitter 20.88 min (*SD* = 21.23), Reddit 34.07 min (*SD* = 44.2), Pinterest 9.53 min (*SD* = 10.64), YouTube 54.20 min (*SD* = 71.32), LinkedIn 9.8 min (*SD* = 11.74), Telegram 14.25 min (*SD* = 21.8), and other not-listed applications 55.8 min (*SD* = 90.96) per day. Descriptive statistics of study variables are listed in Table 1.

### Path analyses

**Fig. 2:**
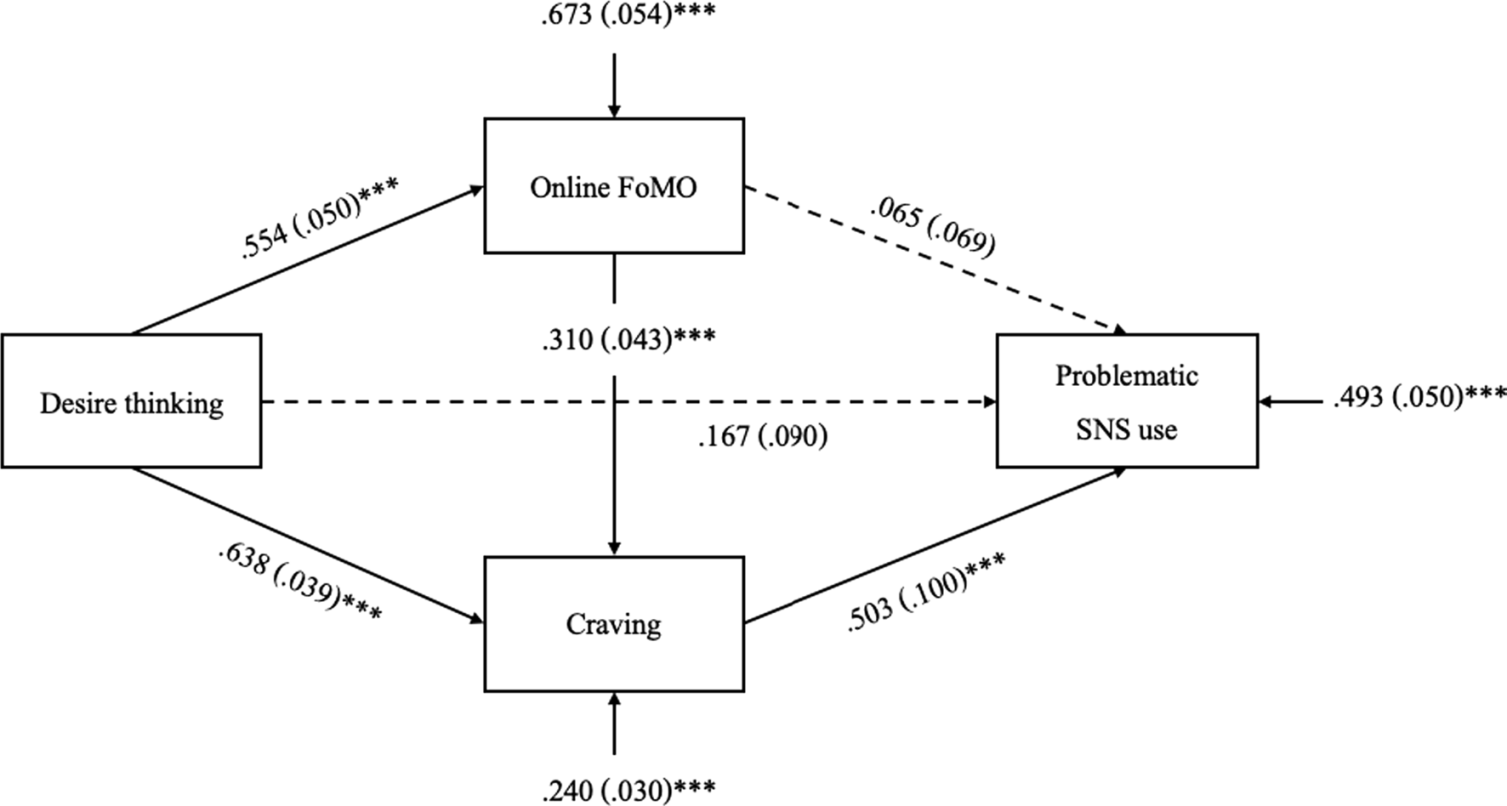
Test of the hypothesized path model*. Note.* Figure shows direct effects with their respective β-weights, standard deviations, levels of significance, and residual variances. ***p < .001

As a requirement for mediation analyses, all variables for the path model were correlated with each other ([53], see Table 1). We controlled all variables for the influence of the experimental randomization preceding the questionnaires. According to significant correlations with age (*p* < 0.05) and significant *t*-tests between gender groups (*p* < 0.05), we controlled desire thinking for the influence of gender; FoMO for the influence of age; and craving and symptom severity for the influences of both. The model fit was excellent with a SRMR = 0.041, RMSEA = 0.067, CFI = 0.997, TLI = 0.969, and χ^2^/df = 1.859. The model explained half of the variance (51%) of problematic SNS use (*R*^*2*^ = 0.507, *p* < 0.001). The direct effect of desire thinking and FoMO on problematic SNS use showed no significant effect. Merely, the direct effect of craving on problematic SNS use was significant (see Fig. 2). Two out of three indirect pathways were significant indicating a partial mediation of craving in the relation of desire thinking and problematic SNS use. Moreover, the effect of desire thinking on problematic SNS use was also significantly mediated by FoMO and craving. The effect of desire thinking on problematic SNS use was not significantly mediated by FoMO solely (see Table 2). Age had a significant effect on craving (β = − 0.116, *SD* = 0.037, *p* = 0.002) and FoMO (β = − 0.118, *SD* = 0.060, *p* = 0.049). The remaining covariate effects were not significant.

**Table 2:** Indirect pathways through the hypothesized model

Path	β	*SE*	*p*
*Indirect 1* Desire thinking → Craving → Problematic SNS use	.321	.067	.001
*Indirect 2* Desire thinking → FoMO → Problematic SNS use	.036	.039	.353
*Indirect 3* Desire thinking → FoMO → Craving → Problematic SNS use	.087	.023	.001

### Ad hoc analyses

**Fig. 3:**
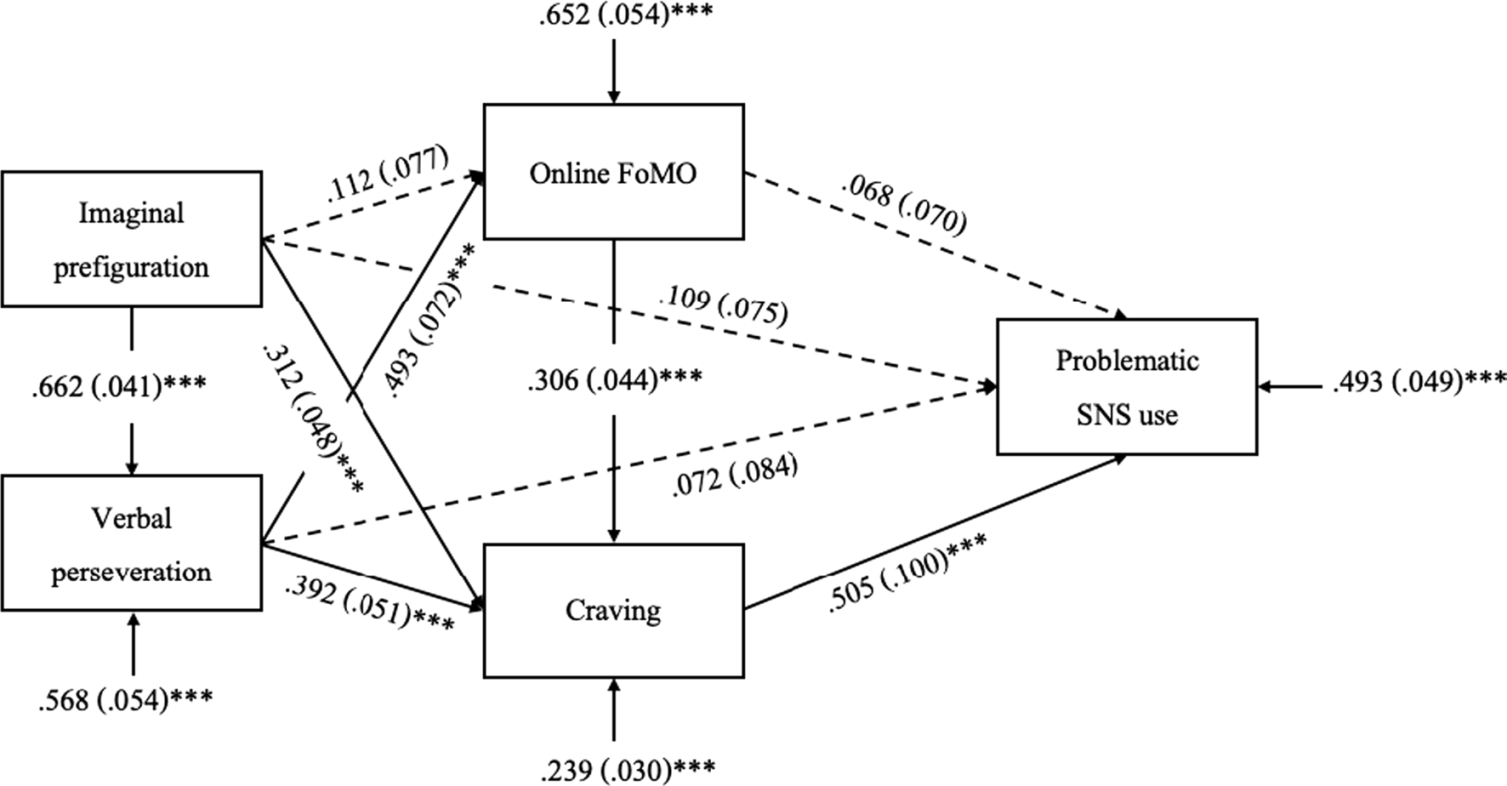
Test of the ad hoc path model. *Note.* Figure shows direct effects with their respective β-weights, standard deviations, levels of significance, and residual variances. ***p < .001

Whereas the primary aim of this study is to investigate whether there is an interaction between desire thinking, FoMO, and craving in predicting problematic SNS use, the literature on desire thinking suggests different roles for the sub-facets of desire thinking (e.g., [54, 55]). Therefore, we report a different version of the model, considering the sub-facets imaginal prefiguration and verbal perseveration as separate predictor variables and according to the metacognitive model of desire thinking [19] where imaginal prefiguration predicts verbal perseveration, and verbal perseveration predicts craving. We controlled all variables for the influence of the experimental randomization preceding the questionnaires. According to significant correlations with age (*p* < 0.05) and significant *t*-tests between gender groups (*p* < 0.05), we controlled imaginal prefiguration for the influence of gender; FoMO for the influence of age; and craving and symptom severity for the influences of both. The model fit was excellent with a SRMR = 0.041, RMSEA = 0.014, CFI = 1.000, TLI = 0.998, and χ^2^/df = 1.038. Direct effects are shown in Fig. 3. Indirect pathways are depicted in Table 3. The strongest direct effects were seen between verbal perseveration and FoMO; and craving and problematic SNS use. Accordingly, indirect effects containing these direct associations were most likely to be significant (see Table 3). The whole indirect effect from imaginal prefiguration, verbal perseveration, FoMO, and craving on problematic SNS use was significant. The only significant effect of control variables was seen for age on craving (β = − 0.116, *SD* = 0.037, *p* = 0.002).

**Table 3:** Indirect pathways through the ad hoc model

Path	β	*SE*	*p*
*Indirect 1* IP → Craving → Problematic SNS use	.157	.039	.001
*Indirect 2* IP → FoMO → Problematic SNS use	.008	.009	.419
*Indirect 3* IP → VP → Problematic SNS use	.048	.056	.393
*Indirect 4* IP → FoMO → Craving → Problematic SNS use	.017	.013	.172
*Indirect 5* IP → VP → Craving → Problematic SNS use	.131	.033	.001
*Indirect 6* IP → VP → FoMO → Problematic SNS use	.022	.023	.336
*Indirect 7* IP → VP → FoMO → Craving → Problematic SNS use	.050	.015	.001

*IP* imaginal prefiguration, *VP* verbal perseveration

## Discussion

The current study investigated a reinforcing loop of desire thinking as stated in the EIT [15] where desire thinking provokes a sense of deficit which was assumed to be constituted of an online-specific FoMO in this study. The interplay of both processes was assumed to increase the craving experience and to contribute to the explanation of a problematic SNS use.

The results of the correlational analyses indicate a strong relationship of desire thinking with problematic SNS use which aligns with previous research in the social media context [28, 30, 46]. This finding underpins the relevance of desire thinking in the context of problematic SNS use. However, this relationship between desire thinking and symptom severity was not significant when including FoMO and craving in the pathway model, thus highlighting that desire thinking is not dangerous per se [19], but rather becomes behaviorally relevant when it leads to dysfunctional cognitions (i.e., FoMO) and an irresistible desire to use SNS (i.e., craving). The same holds for FoMO which is no longer a dominant predictor of problematic SNS use when entered in the path analysis, leaving craving as the only significant direct predictor. Instead, FoMO only contributes to the explanation of problematic SNS use when considered in combination with desire thinking and craving, albeit with a comparatively small effect size.

The results suggest that the interaction of desire thinking, FoMO, and craving might be worth considering when aiming to explain problematic SNS use. Along with our hypothesis, FoMO might indeed be a product of desire thinking in the form of an associated deficit where individuals experience the urge of wanting to know what their peers are doing online. That is, it might be part of desire thoughts around SNS use to elaborate on what might have happened on one’s social media platforms since the last visit and what one might miss when not having a glimpse. The results of this study have further shown that this sequence of cognitive processes might favor the experience of a heightened desire or craving to use SNS. That is, next to existing results on the predictive character of FoMO for problematic SNS use itself [39, 40, 43, 56], FoMO might also take part in the explanation of craving for SNS use. Specifically, FoMO is defined as (1) the apprehension that one is missing out on some rewarding experience and (2) the desire to stay connected, serving as a concurrent strategy to cope this fear [39]. This synergetic dichotomy might be the reason why FoMO seems to be closely related to craving, and why this can also result in problematic SNS use in the long term [10, 26, 39]. More specifically, it is conceivable that the conscious fear to miss out on something is part of the intrusive thoughts as a cognitive part of the craving experience (e.g., [48]). This hypothesis is supported by the results of our ad hoc analyses where the verbal perseveration sub-facet of desire thinking is moderately associated with FoMO whereas the imaginal prefiguration sub-facet of desire thinking is not (see Fig. 3). This, however, does not support our hypothesis that it is the imagination of a future event that might leave a gap between simulation and reality as stated in the EIT [15], but that FoMO might be especially generated from perseverative, verbal desire thoughts. This in turn might amplify the urge to use SNS as the motivational part of the craving experience (e.g., [15]) and may make the frequency of experiencing diminished control, increased priority, and continuation of SNS use despite negative consequences more likely. This implicates that FoMO should be considered when working with clients towards the identification of cognitions that cause their cravings for SNS use. Other than that, the identification and modification of online-specific FoMO as part of the emergence of craving might also prevent phubbing [57-59] which, apart from problematic SNS use being primarily harmful to the individual, also brings along a detriment to social interactions.

Notably, the association between desire thinking and craving in this study is comparatively high (see Table 1) when considering previous studies on this association which averagely report a correlation of .46 among different behavioral and substance addictions (cf., [47, 60-63]—leaving little variance in this study that might be explained by other factors. Specifically, the path models suggest that the strongest indirect pathways in explaining variance within problematic SNS use are pathways via desire thinking and craving (see Tables 2, 3). Comparatively, FoMO contributes a rather small incremental value in explaining symptoms of problematic SNS use. Craving is a construct closely related to diminished control and addictive behaviors [26, 64, 65] and fear of missing out on social-media-related activities might (and should) not be reason enough to assume that someone is developing pathological usage patterns. Rather, studies indicate that an interplay of psychopathologies (e.g., anxiety, depression) and specific cognitions (i.e., use expectancies, boredom proneness) reinforces the relevance of FoMO as potential risk factor of a problematic SNS use [39, 45]. Possibly, desire thinking and craving are less differentiable in the context of SNS use where desire thinking might play a less considerable role since individuals might elaborate on their desire to a lesser extent. Instead, smartphones can be quickly pulled out the pocket as soon as a desire is experienced wherefore elaboration might not be as necessary as if the desired behavior was in the distant future. Incidentally, the same explanation is used to explain smaller effects of SNS-related craving [11]. However, a methodological explanation may be the short time interval in which both questionnaires were answered in the online survey, and the experimental manipulation in the first part of our study which may (despite non-significant differences between groups) have caused carry-over effects. Thus, the long-term question is to what extent individuals really experience an urge or desire compared to what role fears and subjectively experienced (social) needs and deficits play in the development and maintenance of problematic use. Therefore, it seems worthwhile in the future to record the relevance of these fears and deficits. This might be done by recording subjective experiences in daily life through ecological momentary assessments, and through actual experimental conditions (e.g., social exclusion to simultaneously examine whether and how strong the craving for SNS actually is).

This opens discussions for further limitations of this study, such as the cross-sectional design and the use of self-report questionnaires that are prone to response biases. Since we tested a pathway model, causal conclusions cannot be drawn from these results, but rather depict a first insight into associations of the proposed variables. Further, although we measured and discussed problematic SNS use, the sample we recruited was a community sample, thus constituted of individuals who do not necessarily experience frequent symptoms of pathological SNS use, which limits the generalizability of our results, as does our female-dominated sample. However, the linear approach we chose might still be able to provide hypotheses for future research. Although, telling from difference testing and covariates in our model, our data seemed to be untouched by effects of the experimental manipulation, this not necessarily means that there was no effect but rather, that possible effects were outside of our awareness. When considering several study parts separately, future study designs need to consider recreational time-outs to neutralize possible carry-over effects.

## Conclusion

The results of this study let assume that desire thinking may be associated with an activation of online-specific fear of missing out on what happens in one’s social networks. Further, FoMO could be considered an influential factor in the emergence of craving for SNS use. However, FoMO and desire thinking seem to only become behaviorally relevant when considered in combination with craving, underpinning that both concepts are not inherently dysfunctional, but might cause behavioral symptoms when they provoke an irresistible desire to use SNS. Our results not only feed theories considering the etiology and maintenance of addictive behaviors but can be applied in clinical settings to raise awareness among practitioners about the intricate linking of mental processes. As such, clinicians should consider disentangling the subjective craving experiences of individuals with problematic SNS use by specifically asking for mental images around SNS use, a linguistic preoccupation with SNS-related thoughts, and (possibly resulting) fear-based cognitions to implement strategies that mitigate moments of impaired control.

## Data Availability

Data is made available via Open Science Framework (OSF); https://osf.io/xhabz/.

## Abbreviations

ACSID-11: Assessment of Criteria for Specific Internet-use Disorders
CEQ: Craving Experience Questionnaire
DTQ: Desire Thinking Questionnaire
EIT: Elaborated Intrusion Theory (of Desire)
FoMO: Fear of Missing Out
ICD-11: International Classification of Diseases (11th Revision)
I-PACE: Interaction of Person-Affect-Cognition-Execution (Model)
SNS: Social Networking Sites
